# Raman Tweezers as a Diagnostic Tool of Hemoglobin-Related Blood Disorders

**DOI:** 10.3390/s8127818

**Published:** 2008-12-03

**Authors:** Giulia Rusciano, Anna C. De Luca, Giuseppe Pesce, Antonio Sasso

**Affiliations:** 1 Università di Napoli Federico II / Complesso Univ. M. S. Angelo, Via Cinthia 80126, Napoli, Italy; 2 CNISM- Consorzio Nazionale Interuniversitario per le Scienze fisiche della Materia, UdR Napoli, Italy

**Keywords:** Raman Tweezers, thalassemia, biosensor, Hb-related disorders

## Abstract

This review presents the development of a Raman Tweezers system for detecting hemoglobin-related blood disorders at a single cell level. The study demonstrates that the *molecular fingerprint* insight provided by Raman analysis holds great promise for distinguishing between healthy and diseased cells in the field of biomedicine. Herein a Raman Tweezers system has been applied to investigate the effects of thalassemia, a blood disease quite diffuse in the Mediterranean Sea region. By resonant excitation of hemoglobin Raman bands, we examined the oxygenation capability of normal, alpha- and beta-thalassemic erythrocytes. A reduction of this fundamental red blood cell function, particularly severe for beta-thalassemia, has been found. Raman spectroscopy was also used to draw hemoglobin distribution inside single erythrocytes; the results confirmed the characteristic anomaly (target shape), occurring in thalassemia and some other blood disorders. The success of resonance Raman spectroscopy for thalassemia detection reported in this review provide an interesting starting point to explore the application of a Raman Tweezers system in the analysis of several blood disorders.

## 1. Introduction

The field of optical bio-analytical techniques has been dominated, for decades, by fluorescence-based techniques. This high-sensitivity advanced microcopy allows the investigation of biochemical reactions in biological systems, providing high temporal and spatial resolution information [1]. Only recently, Raman spectroscopy has revealed to be an effective tool as a biomedical and bionanalytical technique, promising interesting application even for regular clinical use.

With respect to Fluorescence, Raman spectroscopy is considerably less invasive, as it does not require the use of specific fluorescent label. For this reason, it provides the potential to monitor processes signatures even in a living single cell, which would otherwise be difficult to label without altering their cellular behavior [2-4].

Raman spectroscopy is based on the inelastic scattering of photons by molecular bond vibrations. When a laser beam impinges on a sample, a small portion of the incident photons are inelastically scattered, resulting in a frequency shift. The energy difference between the incident and scattered photons corresponds to a roto-vibrational energy excitation of the interrogated molecular bond. Therefore, the Raman spectrum constitutes an intrinsic molecular fingerprint of the investigated analyte, revealing detailed information on its chemical content, as well as the conformation state of macromolecules [5-9].

Recently, numerous Raman-based techniques have been developed in order to enhance the sensitivity and the spatial resolution of the basic technique. Among these, Surface-Enhanced Raman Scattering (SERS) is a process whereby the Raman scattering is enhanced, up to 14 orders of magnitude, when a Raman-active molecule is close to a roughened metal surface. It has been used for studying the structural arrangement and dynamic evolution of single macromolecules, including protein and DNA [10, 11]. For particular classes of biologically-relevant macromolecules, also Resonant Raman Scattering (RRS) [12], providing an enhancement of the Raman signal up to 6 order of magnitude, has been widely used. This effect occurs when the Raman excitation frequency matches a transition frequency of the molecule [13, 14]. RRS has been used to characterize metalloporphyrins, carotenoids and several other classes of biologically important macromolecules, which have strongly allowed electronic transitions in the visible. This gives rise to a selective enhancement of the Raman spectrum of the chromophoric site (often the active site) without spectral interference from the surrounding protein.

A practical but crucial problem in studying biological systems involves the possible interference of the sample preparation procedure with the systems physiological processes. Most cells can live, grow, and reproduce in liquid growth media, and all these processes involve continuous changes in their biochemical composition, potentially detectable by Raman analysis. However, in this condition, the living cell under study may randomly move away from the confocal excitation volume due to Brownian motion or cell motility. Therefore, the living cells have to be immobilized either physically or chemically, which affects the chemical micro-environment of the living cell and may yield to unknown spurious effects. In this sense, the analytical capability of Raman spectroscopy, as well as most of optical-based analytical techniques, are limited by the inability to manipulate, and therefore analyze the sample under study without fixing it to a substrate. This limitation has been resolved by coupling the Raman spectroscopy with an Optical Tweezers (OT) system. An OT system, first developed by Ashkin in 1986, has been used to trap and manipulate different particles, such as mammalian cells, bacteria, spores and polymer beads, in the dimensional range between 100 nm and tens of microns. [15-17]. This system is based on the generation of an optical potential using a high numerical aperture microscope objective lens, creating an optical gradient. The tight focusing required by both Raman spectroscopy and the optical trap makes combining the two techniques straightforward. The resulting system is indicated as *Raman Tweezers* (RT).

To date, many biological systems, including red blood cells, bacterial spores, liposomal membrane composition, have been studied with Raman Tweezers [18-22]. Notably, Wood *et al* have taken advantage of RT's ability to hold RBCs without any mechanical contact to study their reactions under different conditions [23, 24]. Raman Tweezers has also been employed in the study of disease, for example in discerning healthy from virally infected cells [25-27].

One of the potential advantage of RT as diagnostic tool is the capability not only to characterize a biological system, which allows diagnosis of pathologies, but also to monitor the efficacy of pharmacological treatment in real time. As such, Raman Tweezers promise to be an effective analytical tool not only for research purposes, but also for clinical applications.

The current review emphasizes the potential of Raman Tweezers as a powerful interrogation method for blood diseases. In particular, we used Raman Tweezers to study a specific disease related to oxygenation capability of individual red blood cells (RBCs), known as thalassemia [28, 29].

An erythrocyte is essentially a box, the cell membrane, embedding a viscous solution of hemoglobin (Hb). Hb is the oxygen-carrying metalloprotein of the RBCs. In most humans, the Hb molecule is an assembly of four globular protein subunits (two alpha and two beta chains). Each of these chains is attached to heme, the prosthetic group. In the center of the heme there is one atom of iron that temporally can link an oxygen molecule. In thalassemia, the genetic defect results in reduced rate of synthesis of one of the globin chains that make up Hb. In presence of a reduced synthesis of one of the globin chains, the RBCs are not properly formed and cannot carry sufficient oxygen. The thalassemias are classified according to which chain of the Hb molecule is affected. In α thalassemia, there is an anomalous α globin chains production, while in β thalassemia production of the β globin chains is deficient.

In a previous paper, we have demonstrated that by looking to the Raman spectrum of a single, optically trapped erythrocyte, it is possible to distinguish between normal and β-thalassemic RBCs [28]. In this review, we extend the study to α-thalassemic RBCs, also underlying the potential application of the developed Raman Tweezers system for diagnosis of thalassemia.

## 2. Experimental section

### 2.1. RBCs preparation

Fresh blood was obtained by fingerprint needle prick from several consenting adults. Two women with thalassemia (A and B) were treated: serum ferritin was normal in both. Patient A had alpha-thalassemia and B had beta-thalassemia.

Main blood parameters were:

- A: Hb 11.5 g/dL, RBC 4.600.000/ μL, MCV 73.7 fL, MCH 27.2 pg, MCHC 32.5 %, RDW-SD 39.1; Hb electrophoresis was normal and an alpha-thalassemia genomic pattern was found at molecular level αα/α−;
- B: Hb 10.1 g/dL, RBC 5.190.000 / μL, MCV 64.4 fL, MCH 19.5 pg, MCHC 30.2 %, RDW-SD 35.9; Hb electrophoresis showed HbA2 = 5.2%.

Samples were prepared according to the following procedure: blood (5 μL) anticoagulated by K-EDTA, was diluted in isotonic aqueous NaCl solution (10 mL) and human albumin (0.5 mL, used as membrane protection). A few microliters of this solution were transferred, within a few minutes, to a home-made chamber.

### 2.2. Raman Tweezers apparatus

**Figure 1.**
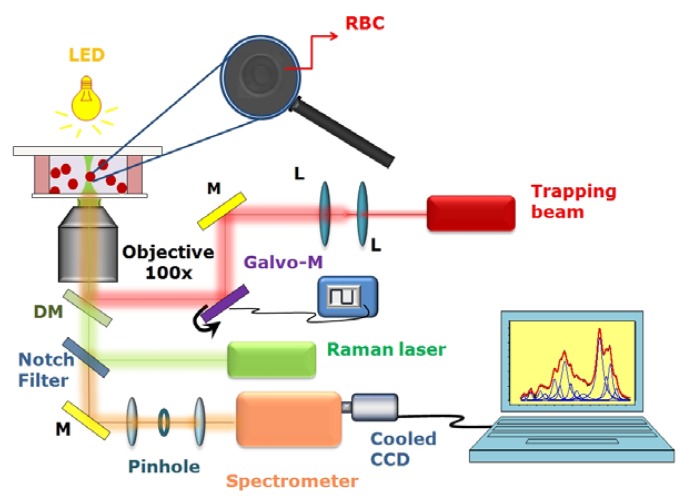
Schematic of our Raman Tweezers system. A Nd: YAG laser at 1,064 nm is used as trapping beam, and a second laser (frequency-doubled Nd:YVO at 532 nm) is used to excite Raman scattering. The two laser beams are introduced into an inverted microscope through a high numerical aperture objective (100 ×). The scattering light from the sample is collected by the same objective and coupled into a spectrometer equipped with a cooled CCD camera. Abbreviation: M-mirror L-lens; DM-dichroic mirror.

The most universal RT system uses two separate beams for trapping and Raman excitation (for example [30, 31]) with a single objective. In such a way, the wavelength and the power of the trapping beam and the Raman probe can be adjusted independently, in order to optimize the system functionality. For instance, to investigate Hb-related diseases such as thalassemia, a Raman probe wavelength in the green region constitutes a good choice, due to occurrence of resonance Raman scattering from the Hb prostheic group. This feature allows the investigation of Hb within erythrocytes without interference by other RBC protein, such as membrane proteins. A schematic describing the set-up of our Raman Tweezers is shown in Figure 1.

A Nd:YAG laser beam (Laser Quantum, Ventus 1064, maximum power 3 W) at 1,064 nm was directed into an home-made inverted microscope equipped with a 100× objective lens (Olympus oil-immersion infinity corrected objective, 1.4 N.A.) to create an optical trap. To optimize the trapping quality, the IR-beam was passed through a telescopic system, which expands the beam diameter up to 12 mm.

Resonant Raman spectra of living RBCs were excited by a frequency-doubled Nd-YVO laser (532 nm; Spectra Physics Millennia Xs). It was mixed to the trapping beam through a dichroic mirror, reflecting near-IR radiation and being transparent to visible radiation. The power of green radiation on the sample was approximately 1 mW. The objective lens used to focus the two lasers onto the sample is also used to collect back-scattered photons. The inelastically-scattered light from the sample passes back along the same optical pathway but through the notch filter. The so filtered radiation was focused through a 50 μm pinhole aperture for confocal geometry and subsequently sent onto the entrance slit of the spectrometer (TRIAX 180, Jobin-Yvon), equipped with a 1,800 lines/mm holographic grating. The confocal detection scheme ensures a spatial resolution of ∼ 0.4 μm in the lateral direction and ∼ 0.9 μm in the axial direction. The Raman radiation was detected by using a front-illuminated charge-coupled device (Pixis 1024, Princeton Instruments, 1,024 × 1,024 pixels), thermoelectrically cooled at -70 °C and placed at the spectrometer exit.

The sample chamber is constituted by two 150 μm glass coverslips (Knittel Glasser, thickness no.1), sealed with parafilm stripes which also act as ∼ 150 μm spacer. The chamber was mounted on a double translational stage: the first provides the coarse sample movement (micrometer translator Newport, HR-13, step size: 2 μm, travel: 13 mm), the second is a piezoelectric stage (Physik Instrumente I-517.3 CL), with a nominal resolution of 2 nm. To allow observation of the trapped particles, the light from a green-filtered LED, focused on the sample by a 10X objective, was used to image the sample onto a CCD camera.

### 2.3. Acquisition of Raman spectra of single optically trapped RBCs and data analysis

A drop of suspended red blood cells in an isotonic solution was placed in the home made chamber. A RBC was positioned near the focus of the trapping beam by moving the manual stage and became stably trapped ∼ 5 μm above the coverslip surface. At this distance, the optical aberrations are reduced and the Raman collection efficiency is maximized. An individual Raman spectrum was acquired on a CCD chip comprised of 1,024 individual channels.

Calibration of the spectrometer was performed by acquiring the Raman spectrum of a trapped polystyrene bead (2 μm diameter, SERVA Electrophoresis), whose Raman peaks positions are accurately known [32]. The final spectral resolution was 2 cm^-1^, as estimated from the polystyrene spectrum by measuring the FWHM of the 1,001.4 cm^-1^ peak.

Raman spectra were collected within the spectral region from ∼600 to 1,800 cm^-1^. This region is known as the spin-sensitive region and provides the most information on hemoglobin oxygenation state [28]. Spectra were background corrected, by subtracting the reference signal, acquired under identical condition but with no RBC placed in the optic trap. A single Raman spectrum was accumulated for 10 s.

To correctly estimate spectral position and intensity of the observed Hb Raman features, the acquired spectral region was fitted with a sum of Lorentzian profiles, each corresponding to an expected Raman feature. All parameters (intensity, spectral position and width) were allowed to vary in the fitting procedure.

### 2.4. Raman imaging

Additional information obtainable by using a RT system relies on the distribution of assigned chemicals within a cell. This kind of application is usually referred as *Raman Imaging or mapping* [33].

**Figure 2.**
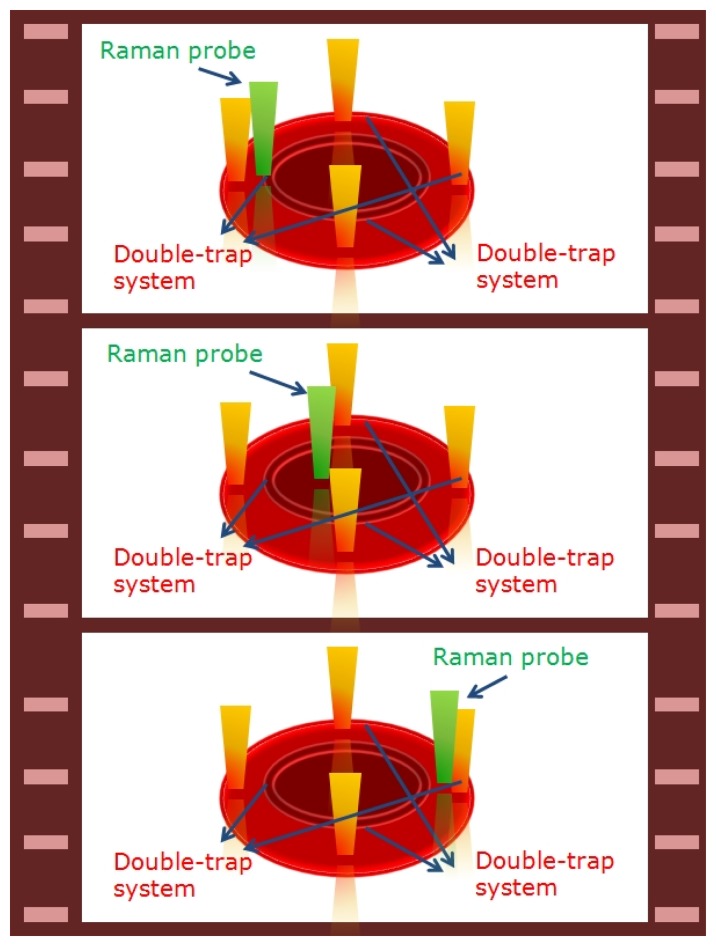
Schematic of the experimental method used for Raman imaging: the four-trap systems fixed the RBC in four points. By applying an offset signal to the galvo-drivers it was possible to scan the sample across the Raman probe.

Raman Imaging in the equatorial plane of RBCs was performed by scanning the trapped cell through the Raman probe (see Figure 2) [34]. At this purpose, two double-trap systems were created by applying a square voltage signal at a frequency of 1 kHz to two galvomirrors (Cambridge Technology Incorporated, mod. 6220), placed on the optical path of the trapping beam. In such a way, the four optical traps shared the beam power and their relative distance was controlled by the voltage signal amplitude. By simply applying an offset signal to the galvomirrors drivers it was possible to scan the RBC through the excitation probe.

## 3. Results and Discussion

### 3.1. Biomolecular characterization of single healthy RBC

**Figure 3.**
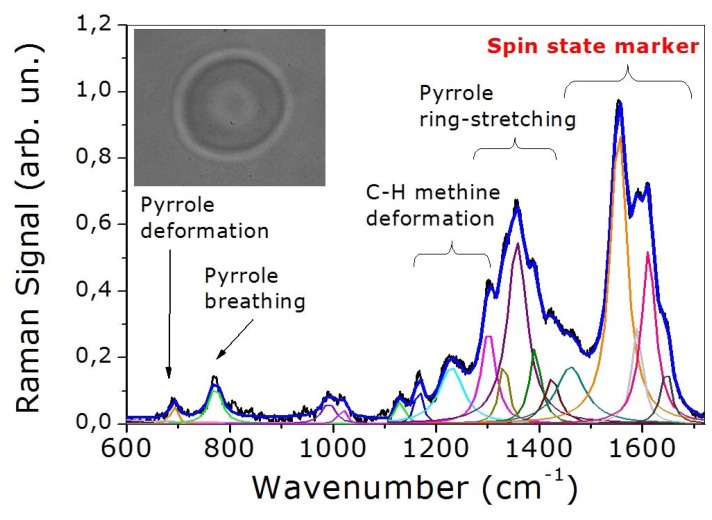
Resonant Raman spectrum of a healthy optically trapped RBC. In the inset we show the image of a trapped erythrocyte.

Figure 3 shows the spectrum recorded from a single healthy RBC. The peaks assignment of this spectrum, which is presented in Table 1, is based on the notation system proposed by Abe *et al.* [35] and by Hu *et al.* [36]. The spectrum exhibits numerous bands associated with the porphyrin macrocycle and proteinaceous component of Hb molecule. It can be divided in essentially four regions. The *core size* or *spin state marker* band region (1,500-1,650 cm^−1^) is comprised of four principal bands appearing at 1,547, 1,588, 1,605 and 1,640 cm^-1^ and assigned to ν_11_, ν_37_, ν_19_ and ν_10_, respectively. The ν_37_ and ν_10_ bands are dramatically modified depending on the spin state of iron or equivalently on the oxygenation state of the analyzed cell. The *pyrrole ring stretching* region (1,300-1,400 cm^−1^) exhibits three principal modes, which can be assigned to pyrrole ring stretching vibrations with different phasing: ν_41_ (1,336 cm^-1^), ν_4_ (1,356 cm^-1^) and ν_20_ (1,397 cm^-1^). The *methine C-H deformation* region (1,300-1,200 cm^−1^) consists of two bands assigned to ν_21_ (1,301 cm^-1^) and ν_13_ or ν_42_ (1,228 cm^-1^). Finally, the *low-wavenumber* region (600-1,200 cm^−1^) contains two principal bands at 760 and 680 cm^-1^ assigned to ν_7_ (symmetric pyrrole deformation mode) and ν_15_ (a pyrrole breathing mode), respectively.

### 3.2. RBCs photo-induced-oxidation

When developing any new analytical tool, it becomes necessary to test possible interference of the analysis procedure with the normal sample functionality. For a laser-based biosensor, this requirement is basically translated in the analysis of possible photo-induced effects. These latter can be tested by observing the Raman bands in the spectral region between 1,500 and 1,650 cm^-1^, since bands are sensitive to photo dissociation [24].

**Figure 4.**
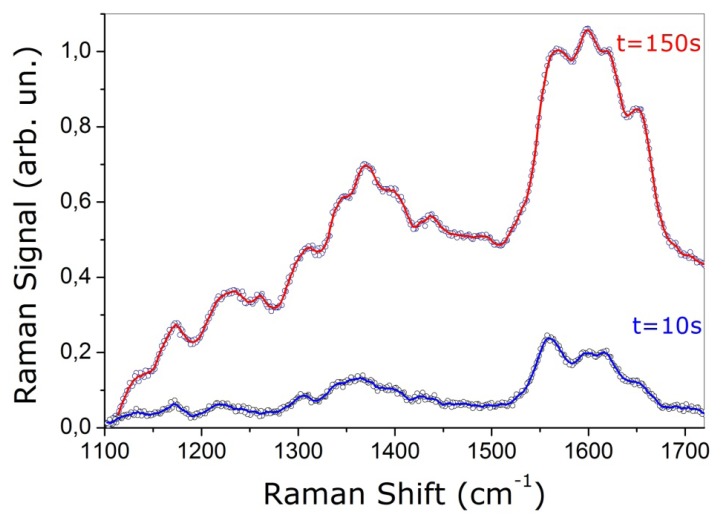
Resonant Raman spectrum of an optically trapped healthy RBC acquired with an integration time of 10 and 150 s.

In our experiment, the photo induced damage of the trapping beam (operating at 1,064 nm) can be minimized by keeping low the power level and by drastically reducing the exposure time. By keeping the trapping laser power level on the sample at ∼10 mW, no noticeable photo induced effect can be observed, even for an exposure time of 100 s [28]. On the other hand, the Raman probe laser at 532 nm can deeply influence the RBC normal functionality. This effect is shown in Figure 4, where we reported the Raman spectra of a healthy single trapped erythrocyte at different integration times (10 s and 150 s). The Raman laser power was set to 0.2 mW. A clear evidence of the photo-damage is the modification of the relative band intensity. In particular, the intensity ratio between the ν_11_ and ν_37_ bands changes such that ν_37_ becomes larger than ν_11_.

**Figure 5.**
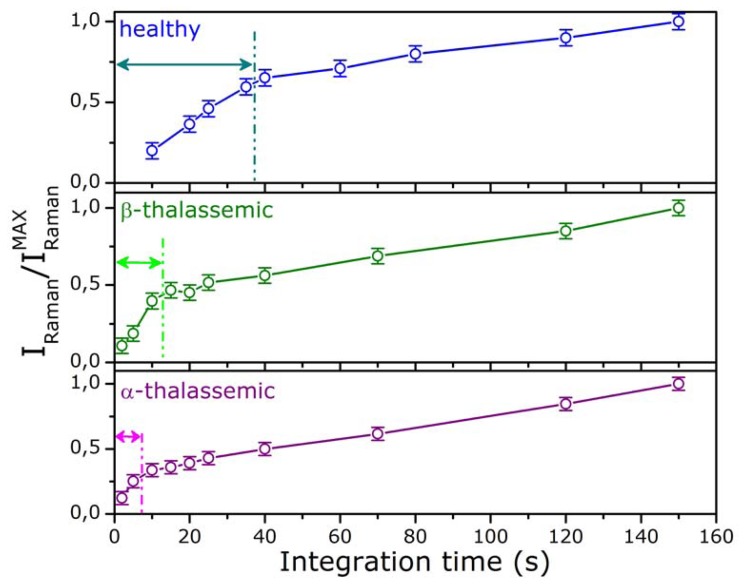
Intensity of the ν_11_ Raman peak, as function of the integration time, with continuous exposure to 532 nm radiation, for normal, β- and α-thalassemic RBCs.

Notably, the influence of the Raman probe is different for healthy and diseased cells. This feature was investigated by monitoring the spectral evolution over time for healthy, α– and β–thalassemic RBCs. The results are depicted in Figure 5, where we reported the intensity of the ν_11_ Raman peak for the three kinds of cells as function of the integration time under continuous exposure to the Raman probe. Each point of Figure 5 represents the average value of ten measurements performed using different cells. Of course, in absence of any photo-damage and instrumental saturation effects, a linear increase of the Raman signal should be expected. This behavior was observed only for reduced integration times: for healthy cells the linear region is limited at integration times less than 40 s, for β–thalassemic RBC at integration times less than 15 s and, finally, this time is reduced to 10 s for α–thalassemic RBC. These outcomes strongly suggest that the photo induced damage can be overcome by setting the trapping and Raman excitation powers on the sample to ∼10 mW and 0.2 mW, respectively, and by reducing the radiation exposure time to 10 s.

### 3.3. Normal versus α- and β-thalassemic RBCs

Current medical approach for diagnosis of thalassemia is based on a number of clinical analysis. A complete blood count, by taking care of hemoglobin concentration (Hb) and Mean Corpuscular Volume (MCV) measurements, is usually the first diagnostic test. In fact, thalassemic subjects have red blood cells that are microcytic (reduced mean corpuscular volume), hypochromic (low hemoglobin value) and have a mild chronic anemia (reduced oxygenation capability), but generally they do not have other symptoms.

**Figure 6.**
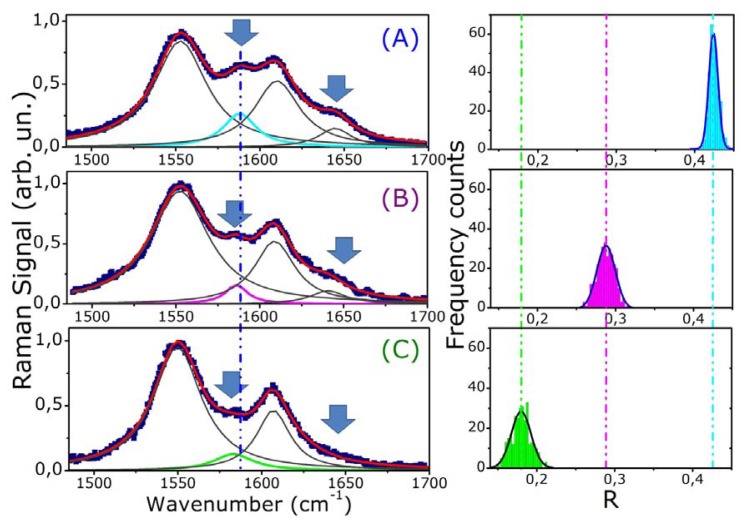
Comparison between the Raman spectra and (right) statistical distributions of the ratio R of the Raman peaks ν_37_ to ν_11_of (A) healthy, (B) α-thalassemic, and (C) β-thalassemic RBC. The arrows in the left part of the Figure indicate spectral features whose intensity changes significantly and the dash-dotted line highlights the observed wavenumber shift of the spectral peaks.

Diagnosis of thalassemia is usually done by exclusion of other causes of anemia. Fractions of hemoglobin A, A2, F, H, E and other variants, as well as serum ferritin are also measured. To get a more detailed frame, some hematologists consider useful to make a microscopic examination of a small amount of blood to analyze the size, shape and color of red blood cells. The final diagnosis is done after globin gene mapping. However, recently new approaches have been proposed. Most of them are based on surface plasmon resonance (SPR) and biosensor technologies [37]. Another quite common technique, used to investigate the reduced oxygenation capability of thalassemic RBCs, is absorption spectroscopy [38]. In this work we demonstrate that the molecular signatures of Hb Raman spectrum and its sensitivity to Hb oxygenation state provide interesting opportunities for developing a new sensor for monitoring blood diseases. This issue has been investigated by comparing the Raman spectrum of healthy and thalassemic cells (α and β kind). In Figure 6 (left part) we reported the spectra acquired by analyzing a single RBC from a normal (A), an alpha- (B) and a beta-thalassemic patient (C). Some important remarks can be done, concerning the relationship of disease with Raman wavenumbers and the observed bands intensity.

The band appearing between 1,580 and 1,588 cm^-1^ assigned to ν_37_ is dramatically modified in both α- and β**-**thalassemic cells. First of all, it is blue shifted on going from normal (1,588 cm^-1^) to α-(1,583 cm^-1^) and β-thalassemic RBCs (1,581 cm^-1^). In addition, its relative intensity decreases for α-thalassemic RBCs and diminishes dramatically for β-thalassemic RBCs. This latter feature is clearly evident also in the ν_10_-band. In fact, the band at 1,640 cm^-1^ is quite small for α-thalassemic cells and completely absent for β-thalassemic one.

As pointed out by Wood *et al.* [24], these strong features can be associated to Hb oxidation state: the porphyrin ring exhibits different symmetries in oxygenated and deoxygenated heme. In such a way, the strength and the spectral position of its vibrations are modified according to the Hb oxidization.

Figure 6 (right part) shows also a statistical analysis on RBCs. For each kind of cell, we estimated the ratio ® of the intensity of the two Raman peaks (ν_37_ to ν_11_), for 300 different cells. In Figure 6, the fits of the experimental distributions with a Gaussian curve are also shown. It is evident from these plots that the distributions are not overlapped within two standard deviations, as highlighted by the dash-dotted lines in Figure 6 (right). Another important observation can be made: distributions of thalassemic cells present much wider spreads around their mean values with respect to that of healthy RBCs. This feature probably is a marker of a higher heterogeneity for RBCs in thalassemic donors.

All these features provide an interesting starting point to explore the application of a Raman Tweezers system in the clinical diagnosis of Hb-related blood disorders. Some considerations can be done, in order to point out some relevant differences and advantages of our approach with respect to traditional investigation tools, as such absorption spectroscopy. First of all, most traditional techniques give information on macroscopic blood samples, containing, therefore, a statistically significant number of cells. This means that they are not able to extol the distribution of oxygen content (or oxygenation capability) between the different RBCs. On the contrary a Raman Tweezers sensor allows analyzing one cell at a time, which enables to reveal differences between single erythrocytes. The disease diagnosis is rapid, taking only few seconds to acquire the Raman spectrum. Finally, no fixative procedure is required, living the sample in physiologically-relevant condition.

### 3.4. Raman imaging of normal and thalassemic RBCs

An observable trait in different hematological diseases, including thalassemia is related to the Hb distribution inside the cell. Although several experimental techniques provide the morphology of single cells, only few of them are able to reconstruct the distribution of an assigned protein within the cell itself. In particular, during recent years, optical phase-based and fluorescence techniques have advanced considerably [39]. However, fluorescence-based techniques often require staining with specific fluorophores, such as GFP, which, in some cases, can affect the normal cellular function. On the contrary, Raman imaging allows the investigation of the distribution of a specific protein, such as hemoglobin, within cells avoiding the use of fluorescent labels.

Herein, we have investigated the Hb distribution inside both healthy- and thalassemic-RBCs. At this purpose, a single RBC was trapped in four points, as explained previously, and scanned across the Raman excitation beam (step-size 0.5 μm). At each step, a spectrum was acquired (integration time for each pixel is 50 ms). By monitoring the ν_11_ peak intensity, we have reconstructed the Hb distribution in the RBC equatorial plane.

**Figure 7.**
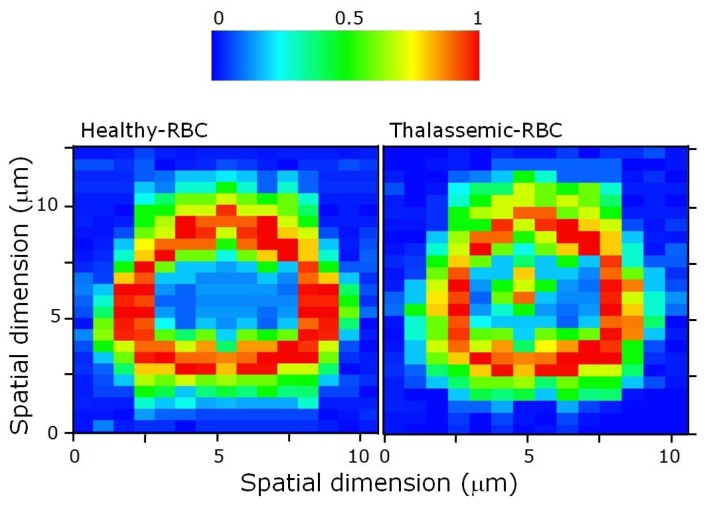
Raman image of a healthy and a thalassemic RBC obtained by reconstructing the Hb distribution along the cell equatorial plane.

Figure 7 shows two separate Raman images of a healthy and a thalassemic cell. The Raman image obtained for a normal RBC, reflecting the Hb distribution within the cell, presents the classical ring-shape. No Hb-Raman signal can be detected in the cell center. An erythrocyte with this normal Hb distribution is ordinarily referred to as discocyte. This distribution is modified in diseased cell. In particular, for a statistically significant number of thalassemic RBCs (∼15%), it is possible to detect Hb also in the middle of the RBC, so that the obtained RBC Raman image resembles a target shape. These RBCs are referred as *target* RBCs or *codocytes*. This effect is associated with a reduced RBC size.

## 4. Conclusions

This work demonstrates that a Raman Tweezers system shows promise of new molecular diagnostic approach in Hb-related blood diseases. At the same time, it describes the potentiality in using this technique for rapid screening of normal and diseased cells, also allowing extolling sample heterogeneity. In particular, by analyzing single erythrocyte of normal, α- and β-thalassemic patients, we demonstrated that the reduced oxygenation capability of diseased cells is translated in a reduced intensity of selected Hb Raman features. This outcome can be used as observable parameter to detect hemopathies. Globally, the proposed method presented some major advantages with respect to traditional diagnosis tools. They include the following: i) only a pinprick of blood is required for the analysis; ii) the diagnosis is rapid, taking only a few minutes for the analysis of a statistically significant number of cells; iii) no staining or fixing to glass substrate is required, allowing RBCs analysis in physiologically-relevant conditions; iv) information on oxygenation state of the RBC can be obtained, at single cell level. More work can be done, in order to develop a fully-automated system for clinical purposes.

## Figures and Tables

**Table 1.** Band position, assignment and local coordinate for normal RBCs recorded using a Raman excitation at 532 nm.

**Band position (cm^−1^)**	**Assignment [36]**	**Local coordinate [36]**
680	ν_7_	*ν(pyr deform)_sym_*
760	ν_15_	*ν (pyr breating)*
993	ν_45_	*ν (C_β_C_1_)_asym_*
1,031	δ(=C_b_H_2_)_4_	*δ(=C_b_H_2_)_4_*
1,134	ν_5_	*δ(=C_b_H_2_)_4_*
1,170	ν_30_	*ν(pyr half-ring)_asym_*
1,228	ν_13_ or ν_42_	*δ(C_m_H)*
1,301	ν_21_	*δ(C_m_H)*
1,336	ν_41_	*ν(pyr half-ring)_sym_*
1,356	ν_4_	*ν(pyr half-ring)_sym_*
1,397	ν_20_	*ν(pyr quater-ring)*
1,429	ν_28_	*ν (C_a_C_m_)_sym_*
1,470	δ(CH_2_/CH_3_)	*δ(CH_2_/CH_3_)*
1,547	ν_11_	*ν(C_β_C_β_)*
1,588	ν_37_	*ν(C_α_C_m_)_asym_*
1,605	ν_19_	*ν(C_α_C_m_)_asym_*
1,640	ν_10_	*ν(C_α_C_m_)_asym_*
